# Comparative Evaluation of Conventional and Digital Techniques for Maxillofacial Prosthesis Fabrication: A Systematic Review of the Literature

**DOI:** 10.7759/cureus.95691

**Published:** 2025-10-29

**Authors:** Meet A Dodia, Nilesh Patel, Chintan Desai, Bedant Chakraborty

**Affiliations:** 1 Prosthodontics, Government Dental College and Hospital, Ahmedabad, Ahmedabad, IND; 2 Prosthodontics, Sidhpur Dental College and Hospital, Sidhpur, IND; 3 Dental Public Health, Tufts University School of Dental Medicine, Boston, USA; 4 Community Dentistry, Eastman Institute of Oral Health, University of Rochester, Rochester, USA

**Keywords:** cad/cam, digital impression, maxillofacial prosthesis, stereophotogrammetry, systematic review

## Abstract

This systematic review objectively evaluates the accuracy and precision of digital impression techniques for maxillofacial prosthesis fabrication and compares them with conventional methods. It also explores advancements such as computer-aided design (CAD)/computer-aided manufacturing (CAM), 3D scanning, and smartphone-integrated stereophotogrammetry.

A comprehensive literature search was conducted across PubMed, ScienceDirect, Wiley Online Library, Cochrane, and Google Scholar until February 2025 following Preferred Reporting Items for Systematic Reviews and Meta-analysis (PRISMA) guidelines. Studies comparing digital and conventional impression techniques for maxillofacial prosthesis fabrication were included. The qualitative analysis was done using the Joanna Briggs Institute critical appraisal tools. Data extraction focused on accuracy, precision, clinical outcomes, and fabrication efficiency. Due to methodological heterogeneity, a narrative synthesis was conducted.

Twenty-five studies met the inclusion criteria. Digital workflows, including intraoral scanning, photogrammetry, and rapid prototyping, demonstrated accuracy comparable to conventional methods. Smartphone-integrated scanning emerged as a cost-effective alternative. However, variations in study designs, scanning techniques, and software limited direct comparisons.

This systematic review compares conventional and digital impression techniques for the fabrication of maxillofacial prostheses. Digital workflows offer improved accuracy and efficiency, but evidence is limited by heterogeneity, small sample sizes, and technical limitations, highlighting the need for further research. Digital impression techniques offer a viable alternative to conventional methods, enhancing accuracy, efficiency, and accessibility, and potentially revolutionizing the fabrication of maxillofacial prostheses, particularly in resource-limited settings.

## Introduction and background

Maxillofacial deformities can be either congenital, resulting from developmental abnormalities, or acquired due to trauma or disease. These defects often cause significant psychological distress for patients, leading to physical, social, and familial challenges. While surgical reconstruction is typically preferred, in many cases, artificial restoration with a maxillofacial prosthesis becomes necessary [1,2]. The Glossary of Prosthodontic Terms (10th edition) [3] has defined maxillofacial prosthesis as “any prosthesis used to replace part or all of any stomatognathic and/or craniofacial structures.” The traditional fabrication of maxillofacial prostheses involves multiple time-consuming steps that can be distressing for patients and depend heavily on the expertise of the manufacturing team, which may include an anaplastologist, maxillofacial prosthodontist, or clinical reconstructive scientist.

In contrast, the digital workflow streamlines the process by capturing defect details in three dimensions. This approach utilizes two types of software: one for processing scan data to visualize the patient’s defect and another for digitally designing the prosthetic component. The first type of software (such as Mimics, 3D Slicer, or equivalent medical imaging tools) is used to process and convert Digital Imaging and Communication in Medicine (DICOM) data from CT or MRI scans into a 3D model, enabling visualization of the patient’s defect. The second type of software (such as Blender, Rhinoceros, or Meshmixer) is then used for computer-aided design (CAD) of the prosthetic component, which can subsequently be fabricated using 3D printing or milling technologies. The purpose of digital scan visualization is to create a virtual representation of the patient and their defect by reconstructing DICOM data into a 3D model using specialized medical software [4].

The digital rehabilitation process consists of four key stages: visualization, design, manufacture, and evaluation. Digital visualization is achieved through various medical imaging techniques, including CT, CBCT, and MRI. Additionally, non-medical methods, including intraoral scanners (IOS), laser surface scanning, and 3D photogrammetry systems, are used to create 3D models. The design and modelling phase is influenced by the data-acquisition method, which plays a crucial role in the digital workflow. This stage allows for detailed sculpting of anatomical structures and shaping virtual clay models into the desired form. Manufacturing is carried out using 3D printing, also known as rapid prototyping, which encompasses techniques such as stereolithography, laser sintering, fused deposition modeling, and inkjet-based systems [5,6].

While the limitations of conventional approaches to the fabrication of maxillofacial prostheses are well-known, the adoption of 3D imaging techniques such as CT scans [7] and MRI [8], despite their precision in providing anatomical details of defects, comes with their own limitations such as high cost, accessibility challenges, and, in the case of CT, exposure to ionizing radiation. To overcome this drawback, novel non-contact 3D digitization techniques, including laser or optical scanning and stereo-photogrammetry, are emerging as safer alternatives [9-11]. These innovative approaches not only eliminate radiation exposure but also enhance 3D modelling for both biomedical applications and artifact reconstruction, making them a superior choice over traditional methods [12-14]. Researchers have also explored the utilization of smartphones for data acquisition and subsequent prosthesis modelling using compatible software [10,15,16].

The prosthetic rehabilitation of maxillofacial defects remains a significant clinical challenge due to the unique anatomical and functional complexities of each patient. Conventional fabrication techniques, including elastomeric impressions, stone casts, and wax patterning, have been widely used but are often time-consuming and technique-sensitive. In recent years, digital workflows incorporating CAD/CAM, 3D scanning, rapid prototyping, and smartphone-integrated stereophotogrammetry have emerged as promising alternatives, offering potential improvements in accuracy, efficiency, and patient comfort. Although multiple studies have compared digital and conventional fabrication techniques, the collective evidence remains scattered and inconsistent across the literature. The objective of this systematic review was to compile and analyze existing comparative studies to evaluate the current evidence on accuracy, precision, clinical outcomes, and fabrication efficiency of digital versus conventional methods in maxillofacial prosthesis fabrication. This synthesis aims to provide clinicians and researchers with a clearer understanding of the present capabilities, limitations, and clinical applicability of digital workflows in maxillofacial rehabilitation.

## Review

Materials and methods

Study Protocol

This systematic review was conducted in accordance with the Preferred Reporting Items for Systematic Reviews and Meta-Analyses (PRISMA) guidelines [17]. The review protocol was prospectively developed, registered, and documented in the PROSPERO database (registration number: CRD42025648290) to ensure transparency and methodological rigor. The focused research question was framed using the population, intervention, comparison, and outcome (PICO) model. The population of interest comprised patients requiring maxillofacial prostheses, specifically individuals with maxillofacial defects or deformities. The intervention or exposure evaluated was the use of digital impression techniques, such as intraoral scanning, 3D photogrammetry, and stereophotogrammetry, as well as other digital workflows, such as CAD/CAM and 3D printing. The comparator was conventional impression techniques, including the use of hydrocolloids, silicone, and polyether impression materials. The primary outcomes assessed included the accuracy and precision of maxillofacial prostheses, patient satisfaction, and quality of life. Secondary outcomes included clinical performance measures, such as retention, stability, and comfort, as well as the time and cost-effectiveness of each impression technique. Based on this framework, the focused research question was defined as, “What is the comparative effectiveness of conventional and digital impression techniques for the fabrication of maxillofacial prostheses in patients with maxillofacial defects?”

Eligibility Criteria

Studies were eligible for inclusion if they were published in English, provided a direct comparison between conventional and digital techniques for the fabrication of maxillofacial prostheses, and addressed non-surgical rehabilitation of maxillofacial defects. Only studies published in open-access journals were considered. Studies were excluded if they provided limited information on data acquisition techniques, focused on surgical rehabilitation, were published in languages other than English, or were reviews, abstracts, editorials, letters to the editor, in vitro studies, or animal studies. Since several subscription-based articles could not be retrieved in full despite exhaustive search efforts, and financial constraints prevented access through paid databases or institutional subscriptions, articles not available through open-access sources were also excluded. Moreover, articles accessible only as abstracts or paywalled summaries were excluded to avoid incomplete data extraction or misinterpretation.

Search Strategy and Selection of Study

A comprehensive literature search was conducted through February 2025 across major databases, including PubMed, ScienceDirect, Wiley Online Library, Cochrane, and Google Scholar. The search was unrestricted by time and language, using Advanced Search Options.

Search String for PubMed

The following search string is used: ("maxillofacial prosthesis"[All Fields] OR "maxillofacial prostheses"[All Fields] OR "maxillofacial rehabilitation"[All Fields]) AND ("CAD-CAM"[All Fields] OR "computer-aided design"[All Fields] OR "computer-aided manufacturing"[All Fields] OR ("3D photogrammetry"[All Fields] OR "stereophotogrammetry"[All Fields] OR "digital impression"[All Fields] OR "intraoral scanning"[All Fields])).

In addition to the electronic database search, a manual search was performed in leading peer-reviewed journals, including The Journal of Prosthetic Dentistry, International Journal of Prosthodontics, The Journal of the Indian Prosthodontic Society, Journal of Oral Biology and Craniofacial Research, and British Journal of Oral and Maxillofacial Surgery. The search was not restricted by date of publication. Additionally, to ensure a focused and efficient search, we limited our Google Scholar search to the top 100 most relevant results, as ranked by Google Scholar's algorithm. The decision to limit the search to the top 100 results on Google Scholar is justified by several factors. Google Scholar's algorithm prioritizes relevant articles based on factors like citation count and author reputation, ensuring that the most relevant studies appear at the top. Additionally, research has shown that the quality and relevance of search results decrease significantly beyond the top 100 results [18]. This approach is also supported by systematic review guidelines, such as the Cochrane Handbook, which suggests limiting searches to the most relevant studies in high-volume settings [19].

**Figure 1:**
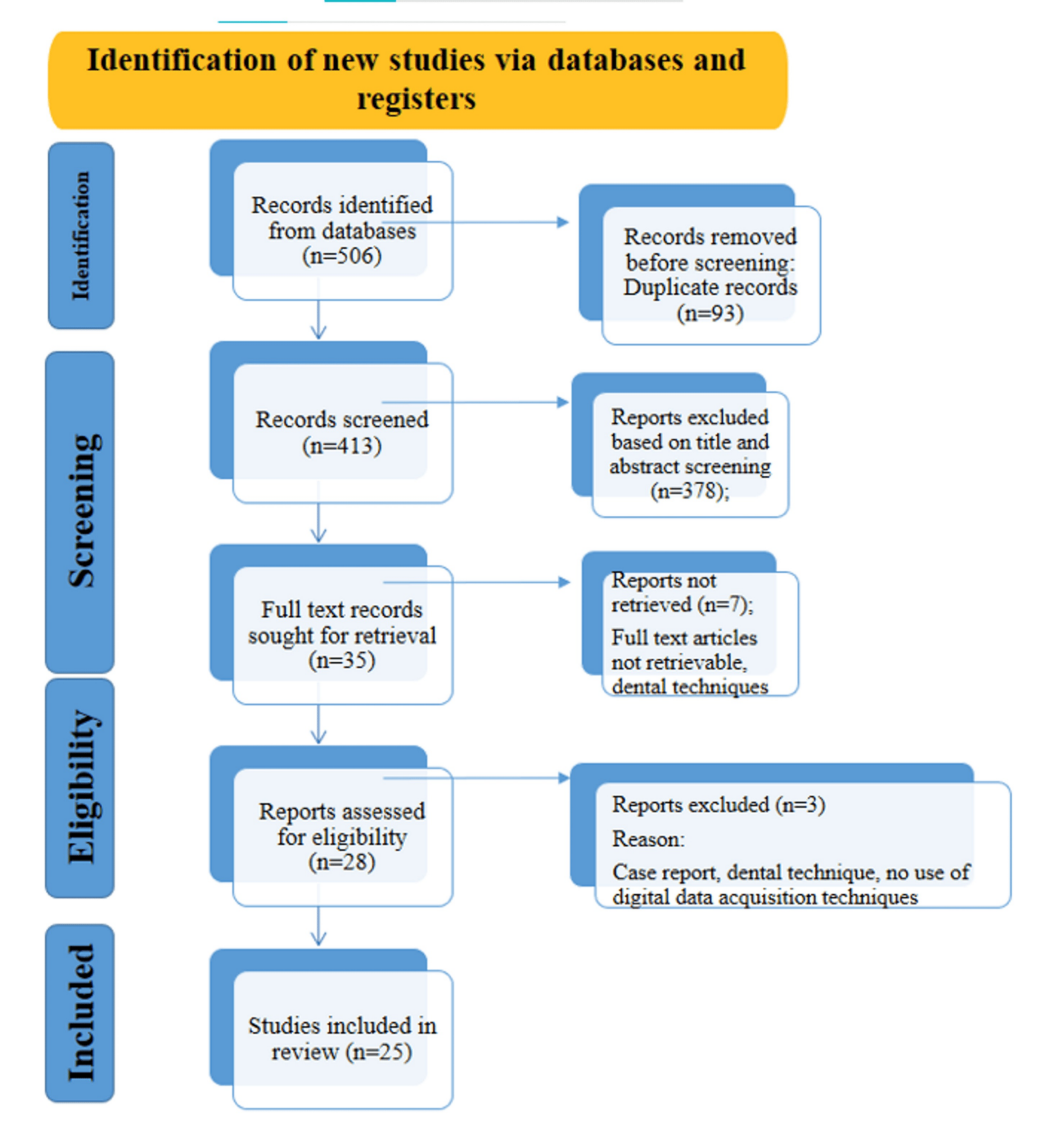
PRISMA flow diagram This figure illustrates the systematic process used to identify, screen, assess eligibility, and include studies in this review, based on PRISMA 2020 guidelines. It shows the number of records identified from databases, duplicates removed, records screened, exclusions (with reasons), and final studies included in the synthesis. PRISMA: Preferred Reporting Items for Systematic Reviews and Meta-Analyses

All the data was imported into reference management software (Zotero, Corporation for Digital Scholarship, Virginia Beach, VA, USA), and duplicates were removed. The detailed flowchart of the literature search following the PRISMA guidelines is presented in Figure 1. Following duplicate removal, two independent reviewers (MAD, NNP) assessed the relevance of each article based on its title and abstract. Articles deemed relevant underwent full-text evaluation. To evaluate the consistency of reviewer decisions, Cohen's kappa statistic was calculated to measure inter-rater agreement during the study selection process.

Data Extraction and Evaluation

Two independent reviewers (MAD and NNP) conducted study selection, with a third reviewer (CD) available to resolve any disagreements and ensure consensus. The full texts of the included studies were retrieved and underwent data extraction using a standardized Excel spreadsheet (Microsoft Corp., Redmond, WA, USA). Extracted data included study demographics, publication year, country, study design, data acquisition methods, software used, prosthesis type, and fabrication methods. The extracted data were subjected to qualitative analysis, and the information on data acquisition, software, prosthesis type, and fabrication methods was tabulated and reviewed to identify the most commonly used methods.

Quality Assessment

Two independent reviewers (MAD, NNP) evaluated the quality of the included studies using the Joanna Briggs Institute (JBI) Critical Appraisal Tools [20], which employed nine questions for quasi-experimental studies, 10 for case series, and eight for cross-sectional studies. The overall appraisal can be calculated as shown in Table 1.

**Table 1:** Application of the JBI critical appraisal tools This table summarizes the critical appraisal process applied to all included studies using the JBI critical appraisal tools, providing an overview of the criteria evaluated. JBI: Joanna Briggs Institute

	Quasi-experimental studies	Case series	Cross-sectional studies
Low risk of bias	≥5 affirmative answers	≥6 affirmative answers	≥5 affirmative answers
High risk of bias	≥5 negative answers	≥6 negative answers	≥5 negative answers
Uncertain risk of bias	≥5 unclear answers	≥6 unclear answers	≥5 unclear answers

Results

Study Selection

A total of 25 articles were included in this systematic review from a total of 506 articles obtained from PubMed, ScienceDirect, Wiley Online Library, Cochrane, and Google Scholar. After excluding duplicates and screening, 35 articles were identified for full-text retrieval, of which 28 were retrieved. Articles not in English were excluded from the study [21-31]; similarly, review articles, such as scoping/narrative reviews, were also excluded from the data synthesis. Furthermore, case reports limited to a single subject [32] and dental techniques [33,34] were not included due to their low level of evidence and the possibility of biased reporting; finally, 25 articles were selected for the systematic review.

Summary

The selected studies (Table 2) encompass a diverse range of methodologies and technologies used in maxillofacial prosthetics, emphasizing advancements in digital tools, imaging, and fabrication techniques. Comparative studies explored the accuracy and efficiency of rapid prototyping, CAD/CAM systems, and intraoral scanning versus conventional methods, highlighting improvements in esthetics, precision, and time efficiency. Analytical and observational studies assessed craniofacial asymmetry, functional outcomes, and the role of multimodal imaging in defect reconstruction. Case series and pilot studies demonstrated the feasibility of emerging techniques such as smartphone-integrated stereophotogrammetry and monocular photogrammetry, offering cost-effective alternatives for facial modeling. Retrospective and controlled clinical trials evaluated the impact of digital workflows on patient outcomes, including speech intelligibility, swallowing, and social acceptance. Despite the growing adoption of digital technologies, some studies noted technical limitations, such as complexity and spatial discrepancies in 3D modeling. Overall, the findings support integrating advanced imaging and fabrication techniques into maxillofacial rehabilitation while underscoring the need for further validation in clinical settings.

**Table 2:** Description of the selected studies This table details the descriptive characteristics of all studies included in the review. Information includes study design, sample size, population demographics, intervention type (digital or conventional impression techniques), comparators, outcomes assessed, and geographic location. ANS: anterior nasal spine, MPT: maxillofacial prosthetists, RP: rapid prototyping, SPG: stereophotogrammetry, FDM: fused deposition modelling, SLS: selective laser sintering, SL: stereolithography, SPINS: smartphone-integrated stereophotogrammetry, IMPRESSeD protocol: improving facial prosthesis construction with contactless scanning and digital protocol, VTK: visual toolkit, DT: digital technologies, OFS: obturator functioning scale, CS: commercial source, OS: open source, DSC: dice similarity coefficient, HD: Hausdorff distance, SPINS: smartphone-integrated stereophotogrammetry, MSA: mesh surface area, VV: virtual volume, IOS: intraoral scanners

Sr. no.	Author	Year	Study design	Population (n)	Intervention/technology	Software/tools	Outcome measures	Results	Conclusion
1	Sykes et al. [35]	2004	Comparative study	n=2 (1 auricular prosthesis, 1 maxillary prosthesis)	RP vs. traditional methods	NA	Accuracy, required time, esthetic appeal	RP showed higher accuracy, better esthetics, and fewer duplication errors	RP offers advantages over traditional methods in facial prosthesis manufacturing
2	Suri et al. [36]	2008	Analytical study	n=15 children (mean age: 11.59 years, repaired unilateral cleft lip and palate)	Craniofacial CT analysis	NA	Craniofacial asymmetry, nasal chamber width, bony alar base support, nasal septum deviation, maxilloalveolar prominence, dental collapse	Significant reduction in nasal chamber width and bony support on the cleft side; deviations in nasal septum, nasal tip, ANS; unilateral dental collapse in 73.3%	Craniofacial CT effectively quantifies structural asymmetries; midfacial regions remain unaffected
3	Hatamleh et al. [37]	2010	Survey study	n=220 (MPT and technologists in the UK)	Use of DT in maxillofacial rehabilitation	NA	Usage trends, advantages, limitations	31% of MPTs used DT; noted benefits: accuracy (23%), planning (23%), record-keeping (14%), color matching (9%), time-saving (9%); limitations: complexity (14%), additional steps (9%)	DT adoption is growing, but technical challenges persist
4	Sun et al. [38]	2011	Case study	n=2 (patients with facial defects)	CAD/CAM system for facial prostheses (structured non-contact light scanning and RP manufacturing)	Microsoft visual C++, VTK	Accuracy, safety, patient comfort, fabrication time	High accuracy and safety, reduced fabrication time, improved patient comfort	Proposed CAD/CAM system is a superior solution for fabricating facial prostheses
5	Jiao et al. [39]	2014	Case series	n=11 (patients with acquired maxillary defects)	CAD and RP for obturator prosthesis fabrication	Mimics materialize NV, GeoMagic Studio 12.0.0	OFS, functional outcomes, social acceptance	Good functional outcomes and social acceptance, OFS scores comparable to traditional methods	CAD/RP technique is a viable method for individualized obturators
6	Artopoulos et al. [40]	2014	Comparative study	n=22 (3D face models from anonymized CT data)	Digital SPG vs. projection moiré profilometry	Di3D FTP-001, MATLAB	Accuracy of 3D surface models	No significant difference between methods (p=0.1882)	Moiré profilometry produces 3D models with accuracy comparable to digital SPG
7	Huang et al. [41]	2015	Case series	n=5 (unilateral maxillectomy patients)	CAD/RP for custom tray fabrication	MIMICS, FREEFORM clay tool	Impression thickness, accuracy, feasibility	Uniform impression thickness, acceptable accuracy (within 1-3 mm) vs. conventional trays	CAD/RP is feasible for fabricating custom trays with consistent accuracy
8	Jiang et al. [42]	2015	Retrospective observational	n=18 (patients with T3-4aN0M0 maxillary sinus cancer)	CAD/CAM hollow obturator prosthesis	MIMICS, Objet Studio, RP with Objet Eden 250TM	Facial measurements, speech intelligibility, chewing, swallowing functions	Significant improvements in speech, facial depression, eyeball prolapse (p<0.05); Swallowing improved at 12 months to level I/II	Simultaneous CAD/CAM prosthesis implantation improves facial profile and function
9	Lincoln et al. [43]	2016	Comparative study	n=6 (reference phantom model)	Facial moulage vs. 3D SPG vs. CBCT	3dMDface, MIMICS 12.1	Accuracy of facial models	CBCT and facial moulage showed the highest accuracy; 3D-SPG models were less accurate	CBCT and facial moulage remain superior in accuracy for facial modeling
10	Ye et al. [44]	2017	Pilot study	n=12 (patients with maxillary defects)	Multi-source data registration (CT + IOS)	Geomagic v2012, Objet 30 PRO	Accuracy of digital casts, clinical effectiveness of prostheses	Digital casts were accurate and clinically effective	3D digital casts of maxillary defects can be generated effectively via multisource data registration
11	Unkovskiy et al. [45]	2019	Comparative analysis	n = auricles of 23 healthy subjects	Comparison of FDM, SLS, and SL for auricular prosthesis replicas	Artec Spider, Artec Studio	Dimensional accuracy and skin texture reproduction	FDM showed superior dimensional accuracy and the best skin surface reproduction	Digital acquisition and CAD postprocessing are crucial for the outcome; FDM is recommended for auricular prosthesis replicas
12	Ross et al. [9]	2018	Comparative study	n = 16 adults (8M, 8F)	Comparison of 3D scanning techniques: Artec Spider, Intel RealSense, and iPhone 7 with photogrammetry	CloudCompare	Scanning time, processing time, accuracy, completeness, resolution, and repeatability	iPhone (30–60 photographs) provided accurate scans with reasonable processing time; Intel RealSense performed poorly	iPhone with photogrammetry is a viable, cost-effective option for 3D scanning of the external ear
13	Jablonski et al. [46]	2018	In vitro	n = 10 plaster casts of oncology facial defects	Multimodal image fusion (SPG and optical structured light scanning)	DI4D, MeshLab	Accuracy, validity, precision	Global mean deviation: 0.22 mm (SD 0.05 mm)	Method is accurate and repeatable for capturing facial defects
14	Matsuoka et al. [47]	2019	Experimental study	n = 7 normal subjects, n = 7 with nasal defects	3D facial scanning and morphing technique	3dMD face System, mHBM	Accuracy of 3D facial expression models	No significant differences between scanned and homologous models	3D facial expression models show high accuracy for fabricating facial prostheses
15	Amornvit and Sanohkan [48]	2019	In vitro	n = 1 master face model, scanned 5× using 4 scanners	EinScan Pro (EP), EinScan Pro 2X Plus (EP+), iPhone X (IPX), Planmeca ProMax 3D Mid (PM)	Rhinoceros 3D Modeling software, Bellus 3D Face app (iPhone X)	Accuracy of 3D face scans	EP+ highest accuracy, followed by EP, IPX, and PM; Fastest scanning: IPX > PM > EP > EP+	Optical scan accuracy depends on the technology used; EP+ recommended for face scanning
16	Farook et al. [49]	2020	Comparative analysis	n = 5 types of facial defects	CS vs. OS software for 3D prosthetic template design	CS: MIMICS, 3matic; OS: Slicer 4.10.2, Meshmixer 2.1	VV, DSC, HD	No significant difference in volume; 67.7% similarity between CS and OS templates (p > 0.05)	Both CS and OS produce similar templates, but with slight differences in landmarks and spatial overlap
17	Matsuo et al. [50]	2020	Pilot study	n = 5 healthy volunteers	Monoscopic photogrammetry with smartphone-recorded monocular video	Xperia X compact, VIVID 9i, RapidForm 2006, AutoDesk Remake	Accuracy of 3D facial models: comparison of nasal component parts	Significant difference in nasal dorsum length; no significant differences in other nasal features	Smartphone video and photogrammetry may be promising for maxillofacial prosthetic rehabilitation
18	Falih et al. [51]	2021	Comparative analysis	n = 2 individuals (1 with facial deformation, 1 healthy)	"+IDonBlender" photogrammetric tool	iPhone 11 Pro Max, Agisoft Metashape, CloudCompare	Geometric error, model resolution, accuracy	Variation: 0.470 mm (coordinates), 0.471 mm (distance)	"+IDonBlender" is a robust 3D modeling tool for maxillofacial applications
19	Farook et al. [15]	2021	Comparative analysis	n = 6 ear casts	Smartphone cameras (OnePlus 5T, iPhone 6s) vs. desktop laser scanner (3D scanner Ultra HD)	GeoMagic Design, ReCap, MeshMixer	Accuracy (surface area, VV, interpoint mismatch, spatial overlap)	No significant differences between smartphone cameras and laser scanner (p > 0.05 for all 4 parameters)	Smartphone cameras can generate 3D ear models comparable to laser scanners
20	Farook et al. [52]	2021	Development and validation study	n = 36 palatal defect models	SPINS vs. intraoral LASER scanners	AutoDesk Recap	MSA, VV, HD, DSC	No significant differences in MSA, VV, HD, DSC between SPINS and laser scan	SPINS is a viable, low-cost method for maxillofacial rehabilitation
21	Beri et al. [8]	2022	Clinical trial protocol	n = 45 patients with craniomaxillofacial defects	Photogrammetry, 3D scanning, conventional impression method	NA	Accuracy of 3D models: method comparison	NA	NA
22	Dohiem et al. [53]	2022	Controlled clinical trial	n = 8 patients with intact right ears	Conventional impression vs. IOS	Geomagic software	3D deviations, accuracy of impression methods	Significant difference in accuracy between conventional and intraoral scans without markers; IOS with markers showed the highest accuracy	IOS provide faster and more accurate impressions compared to conventional techniques
23	Jablonski et al. [54]	2023	Feasibility crossover RCT protocol	n ≤30 patients with orbital/nasal defects	Digital (IMPRESSeD protocol) vs. conventional facial prosthesis manufacturing	Artec Space Spider, Form 3	Feasibility outcomes (eligibility, recruitment, conversion, attrition rates); patient preference, QoL, resource use	Not reported (study protocol)	Not reported (study protocol)
24	Gadallah et al. [55]	2025	Comparative analysis	n = 1 intact ear scanned with 3 IOS	CEREC Primescan, Medit i700, Panda P2	NA	Matching scores of 3D-printed auricular casts	Primescan and Medit i700 better than Panda P2 (p < 0.001)	Primescan and Medit i700 IOS are more accurate for maxillofacial digitization
25	Tsuchida et al. [56]	2023	Comparative study	n = 3 plaster statues	5 handheld-type scanners (Artec Eva, Artec Spider, Bellus 3D FaceApp, SNAP, Vectra H1)	Artec Studio 12 Professional	Scanning time, processing time, and the deviation of the distance between points	Spider: best accuracy (97% within ±1.00% deviation); Eva and Bellus: shortest scanning and processing times	Handheld scanner performance varied; Spider had the best accuracy

Quality Appraisal of Included Studies

Nine quasi-experimental studies were assessed using the JBI checklist for quasi-experimental studies (Table 3). Most demonstrated clear cause-and-effect relationships, consistent treatment protocols, and reliable outcome measurements, with appropriate statistical analysis, indicating a low risk of bias in seven studies. However, two studies (Beri et al. [8]; Jablonski et al. [54]) showed unclear risk due to incomplete reporting of allocation methods, follow-up data, and measurement protocols. The overall evidence from this group provides moderate confidence in supporting the comparative effectiveness of digital versus conventional techniques.

**Table 3:** JBI critical appraisal for quasi-experimental studies This table presents the individual quality assessment results for all included quasi-experimental studies, based on the JBI critical appraisal checklist for quasi-experimental studies. Criteria assessed include study design validity, intervention allocation, outcome measurement consistency, and risk of confounding factors. Each item is scored as “yes,” “no,” or “unclear,” allowing interpretation of overall study quality. JBI: Joanna Briggs Institute

Sr. no.	Author	Year	Cause and effect clarity	Group similarity	Treatment consistency	Control group	Pre-/post-measurements	Follow-up completeness	Outcome consistency	Measurement reliability	Statistical analysis	Overall risk of bias
1	Sykes et al. [35]	2004	Yes	No	Yes	No	Yes	Yes	Yes	Yes	Yes	Low
6	Artopoulos et al. [40]	2014	Yes	Yes	Yes	No	Yes	Yes	Yes	Yes	Yes	Low
9	Lincoln et al. [43]	2016	Yes	Yes	Yes	No	Yes	Yes	Yes	Yes	Yes	Low
13	Jablonski et al. [46]	2018	Yes	Yes	Yes	No	Yes	Yes	Yes	Yes	Yes	Low
14	Matsuoka et al. [47]	2019	Yes	Yes	Yes	No	Yes	Yes	Yes	Yes	Yes	Low
18	Falih et al. [51]	2021	Yes	No	Yes	No	Yes	Yes	Yes	Yes	Yes	Low
21	Beri et al. [8]	2022	NA	NA	Yes	Yes	NR	NR	Yes	Yes	NR	Unclear
22	Dohiem et al. [53]	2022	Yes	Yes	Yes	Yes	Yes	Yes	Yes	Yes	Yes	Low
23	Jablonski et al. [54]	2023	Yes	NR	NR	Yes	NR	NR	Yes	Yes	NR	Unclear

Twelve cross-sectional studies were appraised with the JBI checklist for analytical cross-sectional studies (Table 4). All inclusion criteria were clearly outlined, participant demographics were documented, and standardized measurement techniques were used, with robust statistical analysis. A key limitation was the lack of control for confounding factors across all studies, which weakened interpretability. Despite this, the studies were judged to be at low risk of bias, providing consistent, descriptive evidence on clinical and technological outcomes in maxillofacial prosthetics.

**Table 4:** JBI critical appraisal for cross-sectional studies This table displays the critical appraisal of included cross-sectional studies using the JBI checklist for analytical cross-sectional studies. Evaluation criteria include participant selection, measurement reliability, confounding management, and outcome validity. Scores (“yes,” “no,” or “unclear”) provide a clear overview of methodological rigor and potential sources of bias in these studies. JBI: Joanna Briggs Institute

Sr. no.	Author	Year	Inclusion criteria defined	Study subjects and the setting described	Valid and reliable exposure measurement	Standard criteria used for the measurement of the condition?	Confounding factors identified?	Strategies to deal with confounding factors stated?	Valid and reliable outcomes measurement?	Appropriate statistical analysis used?	Overall risk of bias
2	Suri et al. [36]	2008	Yes	Yes	Yes	Yes	No	No	Yes	Yes	Low
3	Hatamleh et al. [37]	2010	Yes	Yes	Yes	No	No	No	Yes	Yes	Low
10	Ye et al. [44]	2017	Yes	Yes	Yes	Yes	Yes	Yes	Yes	Yes	Low
11	Unkovskiy et al. [45]	2019	Yes	Yes	Yes	Yes	No	No	Yes	Yes	Low
12	Ross et al. [9]	2018	Yes	Yes	Yes	Yes	No	No	Yes	Yes	Low
15	Amornvit and Sanohkan [48]	2019	Yes	Yes	Yes	Yes	No	No	Yes	Yes	Low
16	Farook et al. [49]	2020	Yes	Yes	Yes	Yes	No	No	Yes	Yes	Low
17	Matsuo et al. [50]	2020	Yes	Yes	Yes	Yes	No	No	Yes	Yes	Low
19	Farook et al. [15]	2022	Yes	Yes	Yes	Yes	No	No	Yes	Yes	Low
20	Farook et al. [52]	2021	Yes	Yes	Yes	Yes	No	No	Yes	Yes	Low
24	Gadallah et al. [55]	2025	Yes	Yes	Yes	Yes	No	No	Yes	Yes	Low
25	Tsuchida et al. [56]	2023	Yes	Yes	Yes	Yes	No	No	Yes	Yes	Low

Four case series, evaluated using the JBI checklist for case series (Table 5), reliably reported inclusion criteria, demographics, clinical details, and outcomes. Sample sizes were small, and none documented consecutive recruitment, limiting generalizability. Nevertheless, consistent measurement methods and clear reporting contributed to a low overall risk of bias, supporting these as feasibility-level evidence for innovative digital workflows and imaging solutions.

**Table 5:** JBI critical appraisal for case series This table summarizes the assessment of included case series using the JBI checklist for case series. The criteria assess case selection methods, reporting of demographic and clinical details, completeness of outcome reporting, and study validity. This provides a transparent overview of methodological strengths and weaknesses in the included case series studies. JBI: Joanna Briggs Institute

Sr. no.	Author	Year	Inclusion criteria defined	Condition measured in a standard and reliable way for all participants?	Valid identification methods	Consecutive inclusion	Complete inclusion	Demographic reporting	Clinical data reporting	Outcome reporting	Site demographics	Appropriate statistical analysis	Overall risk of bias
4	Sun et al. [38]	2011	Yes	Yes	Yes	No	Yes	Yes	Yes	Yes	Yes	Yes	Low
5	Jiao et al. [39]	2014	Yes	Yes	Yes	No	Yes	Yes	Yes	Yes	Yes	Yes	Low
7	Huang et al. [41]	2015	Yes	Yes	Yes	No	Yes	Yes	Yes	Yes	Yes	Yes	Low
8	Jiang et al. [42]	2015	Yes	Yes	Yes	No	Yes	Yes	Yes	Yes	Yes	Yes	Low

Certainty of Evidence

The certainty of the body of evidence for each outcome was assessed qualitatively using the JBI critical appraisal tools, applying the checklist most appropriate to each study design (case series, cross-sectional, or quasi-experimental). Each study was evaluated for methodological rigor, risk of bias, adequacy of reporting, and clarity of outcome measurement. Studies with higher JBI quality scores and stronger designs (e.g., randomized or controlled studies) were given greater interpretive weight. In contrast, case reports, technical notes, and studies with unclear methodology were excluded to minimize bias.

Although a formal GRADE (Grading of Recommendations, Assessment, Development, and Evaluation) approach was not conducted due to heterogeneity in study designs, small sample sizes, and variability in outcomes, its principles informed the qualitative assessment. Specifically, confidence in the evidence was based on the consistency of findings across studies, methodological quality, sample sizes, and applicability to the target population. Future systematic reviews with more homogeneous data and meta-analytic synthesis could benefit from a full GRADE evaluation to strengthen evidence grading and recommendations.

Discussion

The prosthetic rehabilitation of ablative defects remains a significant clinical challenge due to the unique anatomical, functional, and esthetic complexities of maxillofacial patients. Each defect presents individualized considerations regarding contour, retention, and tissue mobility, requiring tailored approaches for optimal outcomes. Within this context, the integration of digital technologies has emerged as a transformative tool, offering promising advantages for patient rehabilitation. However, fully digital workflows for maxillary and mandibular intraoral prostheses are still in their early stages of development, with hybrid approaches being the most commonly adopted.

Initially, the focus of digital adaptation in maxillofacial prosthodontics centered on digital image acquisition and model printing, with the definitive cast fabricated via 3D printing, followed by conventional prosthesis construction methods. This semi-digital method served as a transitional phase, bridging traditional craftsmanship with emerging computer-aided workflows. In recent years, advancements in scanning accuracy, modeling software, and affordable additive manufacturing systems have further refined these techniques. The availability of cost-effective open-source design tools has enabled greater clinician participation and reduced dependency on specialized engineering expertise, thereby facilitating the design and fabrication of maxillofacial prostheses [57].

Srivastava et al. [57] conducted a systematic review to assess the feasibility of digital workflows for the fabrication of intraoral maxillofacial prostheses. They concluded that a combination of digital and traditional techniques remains the current standard. They emphasized that the full realization of digital workflows depends on improving data acquisition accuracy and enhancing user accessibility of prosthetic design software. This transition from hybrid to fully digital fabrication mirrors trends in broader prosthodontic practice, where the significant challenges include high equipment costs, steep learning curves, and insufficient longitudinal evidence of clinical reliability. Overcoming these limitations through clinician training, cost rationalization, and cross-disciplinary research collaborations is essential to ensure sustainable integration of digital workflows in everyday clinical practice [58]. However, the present systematic review demonstrates that digital techniques are not merely supplementary but can offer distinct advantages over conventional methods in specific clinical contexts. These include superior dimensional accuracy through CAD-based design, enhanced reproducibility due to automated fabrication processes, reduced patient discomfort by eliminating physical impression materials, and significantly shorter turnaround times from data acquisition to final prosthesis delivery.

The present systematic review further highlights the growing potential of digital techniques to serve as standalone solutions for maxillofacial prosthetic rehabilitation. For instance, Sykes et al. [35] and Sun et al. [38] demonstrated that integrating CAD/CAM and rapid prototyping systems significantly enhances fabrication accuracy, reduces production time, and improves patient comfort compared to conventional methods. Their findings underscore how automation minimizes human-induced dimensional errors and enables more precise replication of complex surface topographies. Likewise, Jiao et al. [39] and Ye et al. [44] reported that CAD-based and multi-source data registration approaches yielded clinical outcomes and esthetic results comparable to or superior to those of traditional workflows. These studies collectively confirm that digitally designed prostheses exhibit superior reproducibility and improved retention fit, particularly in cases of extensive or irregular defects where manual sculpting is technically demanding. The study by Gadallah et al. [55] further demonstrated the clinical reliability of IOS in the fabrication of auricular prostheses. The authors evaluated the dimensional accuracy of three IOS (CEREC Primescan, Medit i700, and Panda P2) for the fabrication of auricular prostheses. The authors reported that Primescan and Medit i700 achieved significantly higher matching scores (p < 0.001) than Panda P2, demonstrating superior accuracy and reliability for maxillofacial applications, such as auricular prosthesis fabrication. The authors also observed that digital scanning produced consistently accurate surface contours and eliminated the need for conventional elastomeric impressions, which are often uncomfortable for patients with sensitive or irradiated tissues. Such findings reinforce the broader conclusion that digital imaging not only enhances fabrication precision but also contributes meaningfully to patient-centered care by reducing discomfort, procedure duration, and anxiety.

Another noteworthy advancement lies in the increasing feasibility of mobile-based data acquisition systems. The current review includes multiple studies [9,15,48,50-52] demonstrating that smartphone-integrated stereophotogrammetry achieves accuracy comparable to that of intraoral laser scanners and traditional impression methods. These developments signal a paradigm shift in prosthetic data acquisition-from reliance on specialized, costly scanning units to highly portable, consumer-grade alternatives. The combined power of modern imaging algorithms, high-resolution smartphone cameras, and open-source 3D modeling software allows clinicians to capture defect morphology directly in a clinical or even home setting. This not only streamlines workflow but also democratizes access, enabling practitioners in resource-limited regions to implement digital prosthetic rehabilitation without significant infrastructural investment.

In addition to technical advantages, digital workflows offer several clinical and operational benefits. Reviewed studies noted reduced chairside time, fewer adjustment appointments, and greater patient satisfaction compared with conventional methods. Digital records facilitate long-term storage, quick retrieval, and seamless data transfer for future modifications or remakes. The adoption of digital platforms also enhances interprofessional collaboration, allowing surgeons, prosthodontists, and anaplastologists to work synchronously on shared virtual models. This collaborative capability improves communication, enhances predictability, and contributes to multidisciplinary care planning, a crucial factor in complex maxillofacial rehabilitation. Despite these advantages, certain limitations persist. The heterogeneity of digital systems, lack of standardized measurement parameters, and variability in scanning accuracy across studies make meta-analysis difficult and limit the generalizability of findings. Moreover, many included studies have small sample sizes and are restricted to case reports or pilot designs, making it challenging to derive definitive evidence regarding long-term clinical performance. There remains a pressing need for standardized validation protocols encompassing dimensional accuracy, mechanical performance, color stability, and patient-reported outcome measures. Until such evidence accumulates, clinicians should exercise caution in replacing traditional methods entirely and instead employ digital workflows as complementary or hybrid tools based on case-specific needs.

From a future perspective, digital maxillofacial prosthesis fabrication is poised to evolve into a fully integrated ecosystem involving artificial intelligence (AI)-assisted design optimization, cloud-based data sharing, and automated additive manufacturing. AI algorithms could assist in contour prediction for defect areas, while machine learning could enhance prosthesis-fitting accuracy using iterative case data. As these technologies mature, accessibility and reliability are expected to increase further, reinforcing digital workflows as the new standard in prosthetic rehabilitation. In summary, this systematic review consolidates evidence supporting the accuracy, efficiency, and patient-centered benefits of digital technologies in the fabrication of maxillofacial prostheses. While the current literature remains heterogeneous, technological progress clearly favors digital adoption. The convergence of affordability, accessibility, and accuracy positions digital prosthodontics not merely as a replacement for conventional techniques but as a transformative innovation capable of redefining clinical practice.

Limitations of Evidence

The evidence base included in this review is limited by the predominance of small-scale studies, most of which use cross-sectional or quasi-experimental designs. Only a few studies featured large participant cohorts or randomized comparisons, and several lacked details on confounder adjustment and completeness of follow-up. Case series, while valuable for feasibility evaluation, inherently carry a higher risk of bias and offer lower levels of evidence. Additionally, inconsistencies in outcome reporting, heterogeneity in digital workflows, and variations in prosthesis types limit the generalizability of findings.

Limitations of the Review

Although this review was conducted using a systematic approach, it still has limitations. Searches were restricted to articles published in English, which may introduce language bias. Grey literature was not included, potentially overlooking emerging digital innovations that have not yet been peer-reviewed. The inclusion of diverse study designs, while necessary for a niche topic, prevented meta-analysis and limited quantitative synthesis. Reviewer bias was minimized by independent data extraction and appraisal, but cannot be entirely excluded. The restriction to open-access journals and the selection of journals were applied to ensure full-text availability, reproducibility, and consistent critical appraisal, given financial and accessibility constraints.

Implications for Practice, Policy, and Future Research

The findings underscore the growing feasibility of fully digital workflows for maxillofacial prosthetic rehabilitation, even in resource-limited settings. Clinicians can leverage smartphone-based imaging and cost-effective design software to reduce costs and enhance accessibility. Policymakers may consider incentivizing digital adoption by integrating affordable scanning technologies and open-source software into training curricula.

Future research should focus on large-scale, randomized controlled trials to evaluate clinical outcomes, patient-reported satisfaction, and cost-effectiveness of fully digital approaches. Standardization of reporting guidelines for digital prosthetic workflows will also improve evidence comparability and accelerate translation into practice.

## Conclusions

This systematic review indicates that digital workflows, including CAD/CAM, 3D scanning, rapid prototyping, and smartphone-integrated stereophotogrammetry, can achieve accuracy and efficiency comparable to those of conventional methods for maxillofacial prosthetic fabrication. Evidence from studies directly comparing conventional and digital techniques suggests that digital approaches can offer advantages in terms of precision, reproducibility, and workflow efficiency. In contrast, conventional methods remain reliable in specific contexts. Smartphone-based stereophotogrammetry further enhances accessibility, allowing clinicians in resource-limited settings to adopt digital workflows without extensive infrastructure. Although heterogeneity in study designs, outcome measures, and fabrication protocols precluded quantitative synthesis, the overall findings support the feasibility of digital techniques as standalone options for maxillofacial rehabilitation. Further research with standardized protocols and larger sample sizes is warranted to strengthen the evidence base and guide clinical implementation.
